# Integration of spatial information in convolutional neural networks for automatic segmentation of intraoperative transrectal ultrasound images

**DOI:** 10.1117/1.JMI.6.1.011003

**Published:** 2018-08-21

**Authors:** Nooshin Ghavami, Yipeng Hu, Ester Bonmati, Rachael Rodell, Eli Gibson, Caroline Moore, Dean Barratt

**Affiliations:** a University College London, UCL Center for Medical Image Computing, Department of Medical Physics and Biomedical Engineering, London, United Kingdom; b University College London, Wellcome/EPSRC Centre for Interventional and Surgical Sciences, London, United Kingdom; c University College London, Division of Surgery and Interventional Science, London, United Kingdom; d University College London Hospitals NHS Foundation Trust, Department of Urology, London, United Kingdom

**Keywords:** convolutional neural networks, prostate cancer, segmentation, transrectal ultrasound, registration

## Abstract

Image guidance systems that register scans of the prostate obtained using transrectal ultrasound (TRUS) and magnetic resonance imaging are becoming increasingly popular as a means of enabling tumor-targeted prostate cancer biopsy and treatment. However, intraoperative segmentation of TRUS images to define the three-dimensional (3-D) geometry of the prostate remains a necessary task in existing guidance systems, which often require significant manual interaction and are subject to interoperator variability. Therefore, automating this step would lead to more acceptable clinical workflows and greater standardization between different operators and hospitals. In this work, a convolutional neural network (CNN) for automatically segmenting the prostate in two-dimensional (2-D) TRUS slices of a 3-D TRUS volume was developed and tested. The network was designed to be able to incorporate 3-D spatial information by taking one or more TRUS slices neighboring each slice to be segmented as input, in addition to these slices. The accuracy of the CNN was evaluated on data from a cohort of 109 patients who had undergone TRUS-guided targeted biopsy, (a total of 4034 2-D slices). The segmentation accuracy was measured by calculating 2-D and 3-D Dice similarity coefficients, on the 2-D images and corresponding 3-D volumes, respectively, as well as the 2-D boundary distances, using a 10-fold patient-level cross-validation experiment. However, incorporating neighboring slices did not improve the segmentation performance in five out of six experiment results, which include varying the number of neighboring slices from 1 to 3 at either side. The up-sampling shortcuts reduced the overall training time of the network, 161 min compared with 253 min without the architectural addition.

## 1 Introduction

Prostate cancer is the most commonly diagnosed cancer in men in the UK, with more than 11,000 deaths per year. Prostate-specific antigen (PSA) testing has been widely used for the diagnosis of prostate cancer, but there is a concern that an over-reliance on PSA as a screening tool could led to an over-diagnosis of low-risk prostate cancer, and subsequent over-treatment of patients with low-to-intermediate risk cancer undergoing conventional radical treatments using radiation therapy or prostatectomy, both of which carry a significant risk of side-effects.^1^ Therefore, both accurate patient stratification and less invasive treatments are critical to improving prostate cancer care.

Recent attempts to address these challenges have led to an emergence of transrectal-ultrasound (TRUS)-guided biopsies and focal therapy techniques that are performed in a highly targeted way. These techniques typically use diagnostic magnetic resonance imaging (MRI) to identify target regions suspected or known to be harboring clinically significant cancer,^2^ overcoming the difficulty associated with reliably distinguishing prostate tumors in a conventional B-mode of TRUS images. However, TRUS remains a safe, low-cost, portable method for guiding the insertion of needles and other instruments into the prostate in real time; and in recent years, a growing number of guidance systems have become available commercially, which spatially register (i.e., align) and fuse MRI and TRUS data to aid targeted needle biopsy. Sankineni et al.^3^ showed that in 26% of patients with prostate cancer, TRUS-MRI fusion-guided biopsy detected the cancer, whereas a conventional, systematic 12-core biopsy did not.

Fully automatic registration of preprocedural MRI with intraoperative TRUS images is a challenging problem due to many factors, such as patient motion, soft-tissue deformation, and marked differences in the image intensity characteristics of the different modalities. Consequently, a feature-based approach is typically employed in commercial and research guidance systems in which the prostate is first segmented in the MRI and three-dimensional (3-D) TRUS images, and the resulting segmentations are aligned using either a rigid or nonrigid (i.e., elastic) registration algorithm. Accurate manual segmentation of the prostate to provide input data when using this approach can be difficult and time-consuming, especially given that process may need to be repeated multiple times during a procedure to account for prostate motion. Moreover, these segmentations are subject to interobserver and intraobserver variability, which can introduce variability in the registration accuracy.

Automating the image segmentation process provides a way to reduce this variability, thereby improving standardization, and reduce the need for extensive manual interaction during a procedure. Furthermore, although fully automated registration methods are starting to emerge that do not require explicit segmentation of the input images,^4^ automated segmentation is still very useful for training, monitoring, and evaluation purposes.

Most prior work on prostate image segmentation has focused on the segmentation of T2-weighted MRI images.^5^^,^^6^ Convolutional neural networks (CNNs) have been shown to achieve high accuracy for the segmentation of these images.^7^^–^^9^ Previous works on automatic segmentation of the prostate from TRUS images have adopted a range of supervised and unsupervised machine learning methods, including texture-feature-extraction methods with support vector machines^10^^–^^14^ and neural networks.^15^^–^^17^ From these last two studies, CNNs have shown to achieve superior performance even for TRUS images compared with other segmentation methods,^18^ and therefore, provide the motivation to use in this work.

In this paper, we evaluate the accuracy of a CNN-based method for automatic prostate segmentation on clinically acquired TRUS images from 109 patients. We have added two modifications to our preliminary work, which was first presented in Ref. 19; first, the incorporation of neighboring slices into the network to be able to use 3-D information for each slice, to take into account 3-D information, which the human observers often consider in the manual segmentation. Incorporating of spatial information has already shown promising results in fetal ultrasound segmentation.^20^ The second modification of this work is an additive up-sampling shortcut architecture as proposed in Ref. 21 for improved training time and performance.

## 2 Methods

### 2.1 Data and Preprocessing

The TRUS images used in this work were acquired as part of the SmartTarget Biopsy Trial,^22^ and consist of 3-D TRUS images of the prostate of 109 patients who underwent targeted transperineal biopsy. For each patient, a continuous rotational 3-D acquisition was used to acquire between 38 and 177 parasagittal slices to cover the prostate gland. During these acquisitions, the TRUS probe was rotated slowly while being held by a stepper cradle equipped with digital position encoders to measure the rotation. After sampling at 3-deg intervals, up to 59 slices per volume were used, leading to a total of 4034 two-dimensional (2-D) slices. The image slices used in this study had a pixel size of 0.18×0.16  mm and an image size of 576×720  pixels, respectively.

The ITK-SNAP software^23^ was used to carry out the manual delineation of the prostate gland (excluding the seminal vesicles). Manual segmentations were performed independently by two observers, with experience in prostate US image analysis (NG/EB). A segmentation of each volume took ∼20 to 30 min to complete. The segmentations from the first observer (NG) were used as the ground-truth both for training of the algorithm and for validating the network segmentation in a cross-validation experiment described in Sec. 2.3, whereas the segmentations from the second observer (EB) were used only for interobserver comparison purposes.

### 2.2 Convolutional Neural Network Architecture

**Fig. 1:**
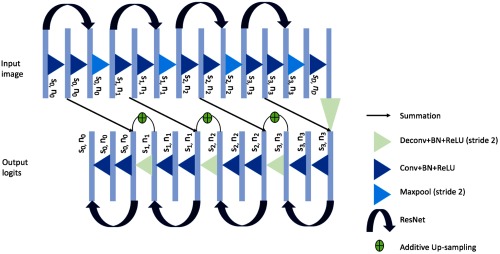
Proposed network architecture.

The algorithm used in this work uses a CNN, which is based on an adapted U-network architecture^24^ proposed in our previous work. The original network takes as input an ultrasound slice of size $S_{0}=[576\times 720]$ and this is propagated to feature maps of the same size and 16 initial channels $n_{0}$ using a convolution (Conv), a batch normalization (BN), and a nonlinear rectified linear unit (ReLU). A kernel size of 3×3 is used for the convolutions. For the present work, for each input image slice, different combinations of the neighboring slices are also considered as summarized in Sec. 2.3. The concatenation between the slice to segment and additional neighboring slices at either side act as 3-D spatial priors for the network. The resulting feature maps are down-sampled to four different resolution levels, where at each level, k=1,…,4, the image size $S_{k}$ is halved and the number of channels $n_{k}$ doubled, meaning following the four downsampling layers the image size is $2^{4}$ smaller than the original size. The down-sampling consists of a troika of Conv, BN, and ReLU, followed by a max-pooling layer with stride 2. This is then followed by a residual network unit (Resnet) block consisting of two Conv layers with BN and ReLU, including an identity shortcut over these layers. The network architecture is shown in Fig. 1.

The up-sampling blocks reverse the down-sampling process using transpose convolution layers with stride 2, replacing the max-pooling layers, and output an image-sized logits layer to represent the segmentation. Reverse Resnet blocks are also included with the addition of additive up-sampling shortcut layers after the transpose convolution layers.^21^ Summation shortcuts are added before each down-sampling block to the output feature maps from each up-sampling block, which is of a compatible size. Summation shortcuts were used in our network instead of concatenation as they have been shown to provide a more smoothly propagated gradient flow and therefore improving the training efficiency,^25^ and provided competitive results in segmenting prostate from MR images.^26^

### 2.3 Training and Validation

The network was implemented in Tensorflow^™^ and trained on a 12 GB NVIDIA^®^ TITAN XP GPU for 10,000 iterations, using the Adam optimizer with 64 images in each minibatch. The results presented here were obtained by minimizing a negative probabilistic dice score that is differentiable with an added $L^{2}$-norm weight-decay on the trainable parameters; the weighting parameter was set to $10^{-6}$.

A 10-fold patient-level cross validation was carried in which images from 11 patients were held out for testing, while the remaining patients were used for training the networks. This was repeated until each of all 109 patients was used for evaluation once. For each automatic segmentation, the largest connected component was chosen to eliminate any isolated foreground segmentations, as a simple postprocessing step. Segmentation metrics were calculated for each fold, by comparing the automatic segmentations to the manually segmented images (ground-truth) using both the binary dice similarity coefficient (DSC) and the boundary distance. The boundary distance was defined as the mean absolute value of the distances between all the points from the automatically segmented boundary and the closest boundary points found on the left-out ground-truth segmentation. Additionally, dice scores were also calculated for 3-D volumes on the patient level. This was computed on volumes reconstructed with the slices from the individual patient. The 3-D DSC is arguably more relevant to the registration application of interest, which requires 3-D prostate TRUS volumes.

Furthermore, to evaluate the impact of the number of adjacent slices, we test the network with different combinations of neighboring slices (on either side), leading to 3-D inputs with 3, 5, and 7 feature maps.

As described in the paper by Karpathy et al.,^27^ there are three different ways of combining the spatial information: early fusion, late fusion, and slow fusion. What we have described in the paper is equivalent to the early fusion pattern. For comparison, we also implemented the slow fusion (using two-adjacent slices on either side) where the neighboring slices are slowly combined through the network, at each layer combining two sets of neighboring slices together and in doing so giving access to more global spatial information as we go to deeper layers, and the late fusion that takes two slices (five slices apart) as two separate networks and only merges these at the fully connected layer, as shown in Ref. 27.

**Fig. 2:**
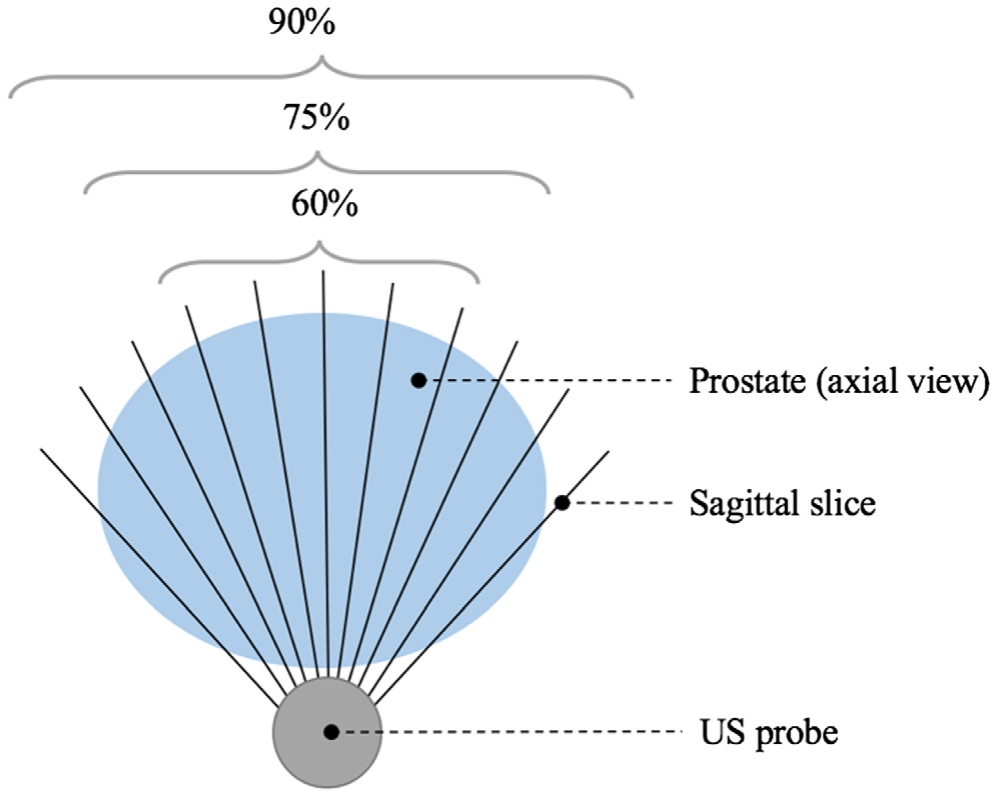
Diagram illustrating the experiment for taking different percentages of midsection prostate slices.

As an additional experiment, the network was also trained by taking a different percentage of the midsection of the prostate for each patient, as shown in Fig. 2. The aim of this experiment was to evaluate the effects of removing slices at the base and apex of the prostate, where the boundary of the prostate is generally considered more difficult to identify.

## 3 Results and Discussion

The computed 2-D and 3-D DSCs and boundary distances averaged over all slices are summarized in Table 1 for the three sets of experiments, using 1, 2, and 3 neighboring slice(s) on each side using the early fusion. From the table, it is shown that taking neighboring slices leads to an improvement in the 2-D DSCs of ∼0.01 on average. Paired Student’s t-tests (α=0.05) were performed to test statistically significant differences between the network without using neighboring slices and those using 1, 2, and 3 slices, with results of p=0.69, p=0.04, and p=0.49, respectively. Increasing the number of neighboring slices seemed to lead to a decrease in the calculated boundary distance, but with p=0.39, p=0.52, and p=0.48 for 1, 2, and 3, respectively, when compared with that without using neighboring slices. Among these results, only one case showed significant difference with p=0.04 when comparing the DSC from the two neighboring-slices case.

**Table 1:** Segmentation metrics obtained from the automatic segmentation results when using different numbers of neighboring slices.

Number of neighboring slices included on each side	2-D DSC	3-D DSC	Boundary distance
None	0.88 ± 0.13	0.88 ± 0.06	1.80 ± 1.68
1	0.89 ± 0.12	0.89 ± 0.05	1.79 ± 2.05
2	0.89 ± 0.13	0.88 ± 0.04	1.77 ± 1.46
3	0.89 ± 0.12	0.88 ± 0.05	1.75 ± 1.77

**Fig. 3:**
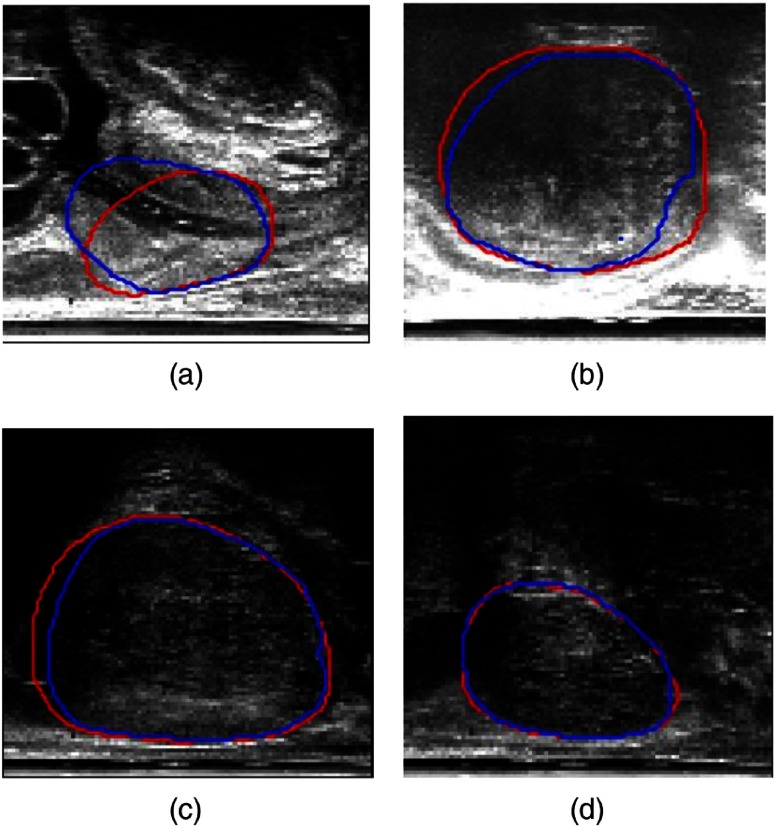
Example comparisons between manual (red) and automatic (blue) segmentations. (a)–(d) Represent the 25th, 50th, 75th, and 100th quantile with DSC of 0.84, 0.92, 0.95, and 0.98, respectively.

Comparison between the automatic and manually segmented images for four example slices (each representing one of the quartiles from the DSC), taking one neighboring TRUS slice on either side, is shown in Fig. 3. The slices shown are chosen with DSC approximately equal to the four quartile values, which are 25th (DSC = 0.84), 50th (DSC = 0.92), 75th (DSC = 0.95), and 100th (DSC = 0.98) quantiles. As illustrated by these examples in Fig. 3, the segmentations were generally more accurate when the prostate boundary was more clearly defined, as seen in slices C and D, respectively. This supports the issue of boundary incompleteness as described in Ref. 11, which is shown to influence the results from the automatic segmentations. This compares well with other works using neural networks for prostate segmentation, reporting a mean DSC value of 0.92,^13^ evaluated on 17 subjects.

The results presented in Table 1 were equivalent to using the early fusion pattern, and as described in Sec. 2.3, the experimental results using two and three adjacent slices on either side, for the slow and late fusion, respectively, are summarized in Table 2. There was no statistically significant difference between the early and slow fusions in terms of the DSC (p=0.34); however, there was a difference in the boundary distance (p=0.03) and for both the DSC and boundary distance when comparing the early and late fusion (p<0.001 and p=0.03, respectively).

**Table 2:** Segmentation metrics obtained from the automatic segmentation results when using slow and late fusion methods.

Fusion method	2-D DSC	3-D DSC	Boundary distance
Slow (two adjacent slices on each side)	0.89 ± 0.12	0.89 ± 0.05	1.68 ± 1.57
Late (three adjacent slices on each side)	0.86 ± 0.12	0.85 ± 0.06	2.15 ± 1.59

**Fig. 4:**
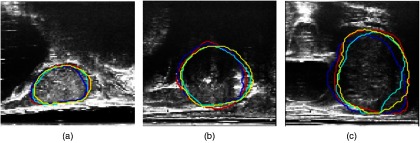
Differences in the automatically segmented prostate when incorporating different numbers of neighboring slices. Manual segmentation (red), automatic segmentation using one adajcent slice (blue), using two adjacent slices (cyan), and three adjacent slices (yellow) overlayed on top of the original prostate slice.

The results of the automatic segmentation for three example slices when taking different numbers of neighboring slices for each patient are shown in Fig. 4. Visually similar contours are observed between each of the automatic segmentations (blue, cyan, and yellow) and the manual ground-truth (red), which is consistent with the results from Table 1. The slice shown in C, however, shows a visual improvement in the yellow contour (using three neighboring slices) when comparing with the ground-truth shown in red, as opposed to the blue and cyan segmentations.

Table 3 shows the segmentation metrics obtained when taking different percentages of slices from each patient (full set of slices, 90% of slices, 75% of slices, and 60% of slices) while incorporating one neighboring slice. There is no statistically significant difference found in the boundary distances (p=0.14, p=0.84, and p=0.67) when using 90%, 75%, and 60% of the middle slices, respectively, nor the 2-D DSC (p=0.17 and p=0.07), when using 90% and 75% of the slices; however, a significant difference is observed using 60% of the slices with p=0.04. This result and the reduction in the standard deviation of both the DSC and boundary distance could suggest that slices near the apex and base of the prostate are indeed more challenging to segment.

**Table 3:** Segmentation metrics obtained from the automatic segmentation results when taking different percentages of middle slices from each patient.

Percentage of slices (%)	2-D DSC	3-D DSC	Boundary distance
100	0.89 ± 0.12	0.89 ± 0.05	1.79 ± 2.05
90	0.88 ± 0.12	0.88 ± 0.06	1.90 ± 1.91
75	0.89 ± 0.10	0.89 ± 0.05	1.78 ± 1.56
60	0.89 ± 0.09	0.89 ± 0.05	1.83 ± 1.42

The addition of the up-sampling shortcuts layer into the network improved the training time from 253 min without any up-sampling shortcuts to 161 min with these up-sampling shortcuts for 10,000 iterations per fold.

### 3.1 Comparison between Different Observers

**Fig. 5:**
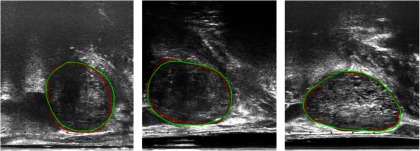
Interobserver segmentation comparisons for three example slices. Good visual agreement is shown between the manual segmentations from the two observers (red and green).

Figure 5 shows examples of manual segmentations from the two different observers, overlaid on top of the original slices for three randomly chosen slices. From the figure, we can see good agreement between the two observers (shown in red and green contours), which also agrees with the computed 2-D DSC between the two observers of 0.92±0.06. These results, together with those reported above, provide a quantitative reference to compare the interobserver and intraobserver variabilities with variability using automatic methods, such as the one proposed in this study, especially under specific clinical context.

### 3.2 Comparison with Other Prostate Segmentation Techniques

We compare the results obtained using our architecture with a state-of-the-art segmentation technique proposed by Anas et al.^17^ Their architecture uses gated recurrent units with the use of residual convolution for improving the optimization of the network. In addition, the authors use a recurrent interconnection between the feature extraction and upsampling branches, which allows the network to incorporate lower-level features in the output segmentation. The mean ± std DSC reported for this paper on 1017 testing slices is 0.93±0.03 and 1.12±0.79  mm for the DSC and boundary distances, respectively. Based on our implementation, their network needs significantly more GPU memory (∼>300 times more based on a single-slice stochastic gradient descent) than the one proposed in this paper.

Previous prostate segmentation techniques using shape models, such as Refs. 11 and 12, report average boundary distances of 0.39±0.05  mm and 1.28±0.03  mm, based on six and eight validation patients’ data, respectively.

## 4 Conclusion

In this paper, we have extended the segmentation CNN we previously proposed in Ref. 19 to incorporate spatial information from neighboring TRUS slices and an additive up-sampling shortcuts in the decoder part of the network. Both qualitative and quantitative results show good agreement between the automatic and manually segmented images when taking a range of neighboring slices, but the inclusion of neighboring TRUS slices with the input image to be segmented was found to make very little or no difference to the segmentation accuracy compared with not including this data for training.

A limitation of this work is that the data used in this study were acquired at a single center, which does not validate its generalization to data from different centers. For future work, the network architecture may be improved specifically for slices near the apex and base of the prostate, which are currently the hardest to segment due to the boundary incompleteness, therefore, by taking this problem into account may improve segmentations results.
